# Dual-Targeted Therapy in HER2-Overexpressing Breast Cancer with Trastuzumab and Novel Cholesterol-Based Nioplexes Silencing Mcl-1

**DOI:** 10.3390/pharmaceutics15102424

**Published:** 2023-10-04

**Authors:** Supusson Pengnam, Praneet Opanasopit, Theerasak Rojanarata, Boon-ek Yingyongnarongkul, Chopaka Thongbamrer, Samarwadee Plianwong

**Affiliations:** 1 Pharmaceutical Development of Green Innovations Group (PDGIG), Faculty of Pharmacy, Silpakorn University, Nakhon Pathom 73000, Thailand; pengnam_s@su.ac.th (S.P.); opanasopit_p@su.ac.th (P.O.); rojanarata_t@su.ac.th (T.R.); 2 Department of Chemistry and Center of Excellence for Innovation in Chemistry (PERCH-CIC), Faculty of Science, Ramkhamhaeng University, Bangkok 10240, Thailand; boonek@ru.ac.th (B.-e.Y.); chopaka.review@gmail.com (C.T.); 3 Pharmaceutical Innovations of Natural Products Unit (PhInNat), Burapha University, Chonburi 20131, Thailand

**Keywords:** HER2-overexpressing breast cancer, lipid nanoparticles, niosomes, siRNA therapy, dual therapy

## Abstract

The challenge in HER2-overexpressing breast cancer therapy lies in creating an effective target therapy to overcome treatment resistance. Monoclonal antibodies and target gene silencing by siRNA are two potential strategies that have been widely developed for treating HER2-positive breast cancer. The siRNA delivery system is a crucial factor that influences siRNA therapy’s success. In this study, lipid-based nanoparticles (cationic niosomes) composed of different cholesterol-based cationic lipids were formulated and characterized for delivering siRNA into HER2-overexpressing breast cancer cells. Niosomes containing a trimethylammonium headgroup showed the highest siRNA delivery efficiency with low toxicity. The myeloid cell leukemia-1 (Mcl-1) siRNA nioplex treatment significantly decreased mRNA expression and breast cancer cell growth. Dual-targeted therapy, consisting of treatment with an Mcl-1 siRNA nioplex and trastuzumab (TZ) solution, noticeably promoted cell-growth inhibition and apoptosis. The synergistic effect of dual therapy was also demonstrated by computer modeling software (CompuSyn version 1.0). These findings suggest that the developed cationic niosomes were effective nanocarriers for siRNA delivery in breast cancer cells. Furthermore, the Mcl-1 nioplex/TZ dual treatment establishes a synergistic outcome that may have the potential to treat HER2-overexpressing breast cancer.

## 1. Introduction

Breast cancer incidence rate has increased in the past few decades and is a major cause of cancer-related death among women globally [1]. Breast cancer is classified into subtypes of triple-negative (basal-like), luminal A, luminal B, and HER2-overexpressing, which are classified based on the expression of estrogen receptors (ER), progesterone receptors (PR), and human epidermal growth factor receptor 2 (HER2) [2,3]. Among the breast cancer subtypes, the triple-negative and ER-/HER2+ subtypes have poor clinical prognoses [4]. Current breast cancer treatments include radiation therapy, chemotherapy, immunotherapy, hormonal therapy, surgery, or a combination of all or some of these [5,6,7]. However, recurrence and treatment resistance are predominant challenges in breast cancer therapy.

HER2 (also known as ERBB2/neu) is a tyrosine kinase transmembrane receptor of the plHER family (EGFR/HER1, HER2, HER3, and HER4), which is amplified and/or overexpressed in various cancer types, including early-stage breast cancer. HER2 is involved in regulating the proliferation, differentiation, and survival of the cell via the activation of downstream RAS/MAPK and PI3K/AKT signaling pathways [8,9]. In recent decades, HER2-targeted therapies have been beneficial for patients with HER2 overexpression/or amplification. Therefore, HER2 inhibitors have been developed for treating patients with breast cancer. Trastuzumab (TZ) is the first anti-HER2 monoclonal antibody and was approved in 1998 for breast cancer therapy both in monotherapy and adjuvant/neoadjuvant therapy with chemotherapy and/or surgery [10,11]. TZ binds specifically to HER2 and results in HER2 signal disruption via several mechanisms, including antibody-dependent cellular cytotoxicity activation, HER2 dimerization, internalization inhibition, RAS/MAPK and PI3K/AKT signaling pathway inhibition, and vascular endothelial growth factor reduction [12,13,14]. Although TZ has been widely used in HER2-overexpressing breast cancer treatment, inherent and acquired TZ resistance still occurs, leading to the progression and recurrence of HER2-positive cancer. The resistance mechanisms of TZ include mutation of HER2, the overexpression of other tyrosine receptors (IGF-IR or c-MET), an alteration of the phosphatidylinositol 3-kinase (PI3K) pathway, loss of the tumor suppressor PTEN, and low immune response [14,15,16]. Moreover, the patient’s cardiac function must be monitored during TZ treatment since it has been reported to cause cardiotoxicity [17]. Therefore, alternative strategies for HER2-overexpressing breast cancer, such as new potent HER2 target compounds, targeted pathways, or dual therapy, need to be developed to improve clinical outcomes and overcome resistance.

Myeloid cell leukemia-1 (Mcl-1) is a pro-survival protein in the BCL-2 family, which binds to pro-apoptotic proteins (BAK/BAX) via the BH3 domain and results in increased cell survival and inhibition of programmed cell death. The up-regulation of Mcl-1 is usually found across a wide type of cancers, including breast cancer, and correlates to poor clinical prognosis [18,19,20]. Mcl-1 expression is elevated during chemotherapy and may be involved in cancer treatment resistance [21]. HER2-overexpressing breast cancer has high Mcl-1 expression; therefore, it is a fascinating therapeutic target for HER2-positive breast cancer [18,22,23]. Several strategies have been developed to target Mcl-1, including Mcl-1 inhibitors (BH3-mimetics drugs) and Mcl-1 silencing by small interfering RNAs (siRNA) [24,25]. RNA interference (RNAi) was first discovered in the nematode worm *Caenorhabditis elegans* [26] and is a post-translational process that degrades target mRNA via double-stranded RNA. This process is initiated by siRNAs, which are 19–23 bps double-strand RNA molecules. Briefly, siRNA is loaded onto the RNA-induced silencing complex and unwound to remove the passenger strand by the Argonaute-2 protein. Then, the RNA-induced silencing complex mediates the sequence-specific binding of the guide strand to the targeted mRNA, which leads to mRNA cleavage and gene silencing [27]. Since genetic alterations are usually involved in cancer progression, RNAi has the potential for use in various cancer treatments, including breast cancer.

The major challenge of siRNA therapy is its delivery to the target site. An effective delivery system should be biocompatible, display non-immunogenicity, protect siRNA through circulation, avoid renal clearance, and reach the target site [28]. There are several siRNA delivery systems, including viral and non-viral systems [29,30]. Niosomes are non-viral lipid-based nanocarriers that are widely used in nucleic acid delivery. They are similar to liposomes in that their phospholipids are replaced by nonionic surfactants [31]. Niosomes have multiple advantages over liposomes, such as low preparation costs and higher chemical stability [32,33]. Cationic niosomes are usually composed of three constituents: (1) a nonionic surfactant; (2) a helper lipid; and (3) a cationic lipid [34]. Variations in these constituents and the method used for preparation influence niosome properties such as size, homogeneity, and cationic niosome stability [35,36]. Nonionic surfactants in niosome formulations (e.g., polysorbates (Tween^®^) or sorbitan monolaurate (Span^®^)) enhance the stability and rigidity of niosomes. The steric hindrance of the hydrophilic groups in nonionic surfactants may inhibit aggregation, which results in the increased stability and rigidity of lipid particles [37,38]. Once niosomes are transfected into cells, they are preferentially internalized by caveolae-mediated endocytosis or micropinocytosis, which can overcome lysosomal degradation [39,40]. Additionally, niosomes prepared from an increased amount of hydrophilic surfactant (such as Tween20) enhance the cellular internalization performed by caveolae-mediated endocytosis, whereas the more lipophilic niosomes tend to internalize via clathrin-mediated endocytosis [41].

Cationic lipids provide a positive charge on the surface of niosomes, which is important for interaction with the negative charge of the phosphate groups on the nucleic acid. Cationic lipids basically consist of three parts: a hydrophobic tail, a linker, and a hydrophilic headgroup [42]. The cholesterol-based cationic lipid, DC-Chol, was first developed and incorporated into liposome formulation by Gao and Huang in 1991 [43]. Different cholesterol-based cationic lipids have been designed for nucleic acid delivery due to their advantages over aliphatic tails, such as improvements in nanoparticle rigidity, biocompatibility, and the ability to fuse with the cell membrane [44]. It has been reported that the different hydrophilic headgroups of cationic lipids affect the nanoparticle properties and transfection efficiency [45,46,47]. Lipid nanoparticles (i.e., liposomes) composed of divalent lysine diamine-conjugated cholesterol-based cationic lipids with different cationic headgroups (e.g., ammonium, trimethylammonium, and guanidinium) have differing properties and transfection efficiencies in various cancer cells [48]. A previous study reported that liposomes formulated from DOPE and cholesterol-based cationic lipids with a trimethylammonium (6a) or tetramethylguanidinium headgroup (4a and 8a) are effective cationic lipids for DNA delivery in human embryonic kidney cells (HEK293) [49]; however, composition variation in the formulations used may affect the properties and transfection efficiencies of the lipid-based carriers.

Herein, niosomes were prepared from different cholesterol-based cationic lipids and a nonionic surfactant (Span20). These cationic niosomes were characterized and utilized for siRNA delivery to breast cancer cells. Gene silencing was targeted at the anti-apoptotic gene, Mcl-1, and cell-growth inhibition was evaluated. Mcl-1 nioplexes were combined with TZ for dual therapy in HER2-overexpressing breast cancer cells. The cell-growth inhibition, apoptosis, and mRNA expression levels after single and dual treatments were determined. The synergistic effect and dose prediction of the dual-targeted therapy were evaluated with computer modeling software (CompuSyn version 1.0).

## 2. Materials and Methods

### 2.1. Materials

**Figure 1:**
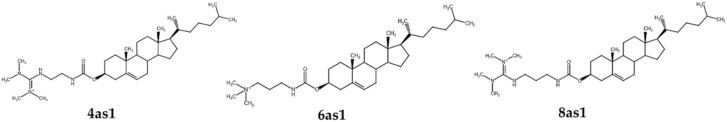
Structure of the cationic lipids used in this study.

Cholesterol-based cationic lipids were synthesized and characterized according to a previous study [49] and kindly provided by Dr Boon-ek Yingyongnarongkul. The cholesterol-based cationic lipids used in this study were coded as 4as1, 6as1, and 8as1. Their structures are provided in Figure 1. Span20 was purchased from Sigma Aldrich^®^ (St. Louis, MO, USA). MTT (3-(4,5-Dimethylthiazol-2-yl)-2,5-Diphenyltetrazolium Bromide) was purchased from Invitrogen™ (Thermo Fisher Scientific, Waltham, MA, USA). The control scramble siRNA (siNT) and siRNA targeting apoptosis-related proteins Mcl-1 (siMcl1) was obtained from Ambion™ Silencer™ siRNA (Cat# 4390824); (Thermo Fisher Scientific, Waltham, MA, USA). The Alexa Fluor 488-labeled siRNA (siAF488) was obtained from Qiagen (Santa Clarita, CA, USA). Annexin V-APC and SYTOX™ Green were purchased from Invitrogen™ (Thermo Fisher Scientific, Waltham, MA, USA). All cell-culture reagents and media were purchased from Gibco BRL (Rockville, MD, USA). The BT474 and MCF-7 cell lines were obtained from the American Type Culture Collection (Manassas, VA, USA). They were cultured in Dulbecco’s Modified Eagle Medium supplemented with 10% fetal bovine serum and 1% penicillin–streptomycin according to the recommended protocol.

### 2.2. Cationic Niosome Preparation

Cationic niosomes were formulated using the thin-film method followed by probe sonication. The Span20 and cationic lipids were dissolved in a methanol:chloroform mixture (1:2 *v*/*v*). The niosomes were prepared by mixing the Span20 solution and cationic lipid solution at the Span20/cationic lipid molar ratios of 2.5:1.5, 2.5:2.5, and 2.5:3.5. The thin film was generated by evaporating the solvent under nitrogen gas and kept in a desiccator overnight. The thin film was hydrated with a warmed Tris buffer (pH 7.4) and incubated at 37 °C for 5 min. Niosome particle size was reduced via probe sonicator for 30 min with 2 cycles at 4 °C. The niosome suspension was then centrifuged at 15,000 rpm for 15 min to eliminate the large particles. The prepared niosomes were stored at 4 °C until use.

### 2.3. Cationic Niosome Characterization

The hydrodynamic size distribution and zeta potential of the prepared cationic niosomes were determined via dynamic light scattering and electrophoretic mobility using Zetasizer Nano ZX (Malvern Panalytical, Malvern, UK). The niosome suspensions were diluted 100 times with sterile water before the size and zeta potential were measured. Measurements were performed in triplicate, and the data were presented as the mean ± standard deviation (SD).

Cationic niosome morphology was evaluated using a transmission electron microscope (TEM) (Tecnai™ G2 20, FEI Company, Hillsboro, OR, USA). The microscope was operated at an acceleration voltage of 80 kV. The niosome suspension was dropped onto the carbon-coated TEM grid and stained with 1% uranyl acetate before photographing.

### 2.4. Cytotoxicity of Niosomes

The toxicity of the prepared cationic niosomes was evaluated by MTT assay. Briefly, BT474 cells were seeded on a 96-well plate at 10^4^ cells per well and incubated at 37 °C and 5% CO_2_ for 24 h. The cells were treated with a cationic niosome suspension at various concentrations up to 500 μg/mL and incubated under normal conditions for 72 h. The treated media were replaced by fresh culture media containing MTT at the final concentration of 1 mg/mL, then incubated under normal conditions for 2 h. The formazan crystals were dissolved in DMSO, and the solution absorbance was measured at 550 nm using a VICTOR Nivo^®^ Multimode Microplate Reader (PerkinElmer, Waltham, MA, USA). Equation (1) was used to calculate the percentage of cell viability, and the IC50 was computed from the plot.

(1) $$\mathrm{Cell viability}\;(\%)=\frac{\left(\mathrm{Abs of treated cells-Abs of DMSO}\right)}{\left(\mathrm{Abs of untreated cells-Abs of DMSO}\right)}\;\times \;100$$

### 2.5. Nioplexes Preparation and Characterization

To formulate the siRNA nioplexes, the siRNA (10 pmol) was mixed with the cationic niosome suspension at various weight ratios. The mixtures were incubated at ambient temperature for 30 min to allow for self-assembly nioplex formation. The nioplex properties were characterized by measuring their size and zeta potential using the same protocol discussed above. Measurements were performed in triplicate, and the data were presented as the mean ± SD.

### 2.6. Cellular Uptake Ability of Nioplexes

#### 2.6.1. Flow Cytometry

The ability of cationic niosomes to deliver siRNA into cells was quantified using a flow cytometer (Attune™ NxT Flow Cytometer, Thermo Fisher Scientific, Waltham, MA, USA). In short, BT474 and MCF-7 cells were seeded in 24-well plates at 3 × 10^4^ cells/well and incubated under normal conditions for 24 h prior to transfection. The nioplexes were prepared via self-assembling siAF488 (30 pmol/well of siAF488), and niosomes with various weight ratios were transfected into the cells. After 24 h of transfection, the cells were washed with phosphate buffer saline (PBS), then trypsinized and fixed with 4% formaldehyde solution. The mean fluorescence intensity (MFI) and percentage of AF488 positive population were observed compared with non-transfected cells as a control. The autofluorescent cell population was compensated for by gating the cell population at 1% non-transfected cells.

#### 2.6.2. Fluorescence Imaging

Fluorescence imaging was conducted to confirm the cellular uptake of the nioplexes in the BT474 and MCF-7 cells. For this purpose, the cells were seeded onto the glass coverslip in 24-well plates at 3 × 10^4^ cells/well and incubated for 24 h before adding siAF488 nioplexes. After 24 h of transfection, the cells were washed twice with PBS, and the cytomembrane and nucleus were stained with wheat germ agglutinin tetramethylrhodamine conjugate (WGA-TC) and Hoechst 33342 trihydrochloride for 15 min. The stained cells were washed with PBS and fixed with a 4% formaldehyde solution. The glass coverslips were mounted onto the glass slides with ProLong™ Diamond Anti-fade Mountant (Thermo Fisher Scientific, Waltham, MA, USA). The fluorescence images were obtained using an Apotome Fluorescence Microscope (ZEISS, Jena, Germany).

### 2.7. Cell-Growth Inhibition by the Single Treatment and Dual-Targeted Approach

#### 2.7.1. Cell-Growth Inhibition by Mcl-1 Nioplexes

The cell-growth inhibition by the single Mcl-1 nioplexes was performed via MTT assay. In short, BT474 cells were cultured in a 96-well plate for 24 h before transfecting with the nioplexes. The Mcl-1 nioplexes were formulated by mixing Mcl-1 siRNA (0.1 μM/well) with the niosomes at various niosome:Mcl-1 siRNA weight ratios. The cells were transfected with the nioplexes and incubated under normal conditions for 72 h. The transfecting media were replaced with fresh media containing MTT and further incubated for 2 h under normal conditions. The MTT media were removed, and the formazan crystals were dissolved by DMSO (100 μL/well). Absorbance was detected at 550 nm using a microplate reader. The percentage cell-growth inhibition was calculated by Equation (2), and the siNT nioplex transfected group was used as a control.

(2) $$\%\;\mathrm{Growth}\;\mathrm{inhibition}=\frac{\left({\mathrm{Abs}}_{\mathrm{cont}.}^{\#}\;-\;{\mathrm{Abs}}_{\mathrm{treated}}^{\#}\right)}{{\mathrm{Abs}}_{\mathrm{cont}.}^{\#}}\;\times \;100$$

where Abs^#^ is the adjusted absorbance (Abs of formazan solution—Abs of DMSO).

#### 2.7.2. Cell-Growth Inhibition by Dual-Targeted Approach

The cell-growth inhibition by the dual-targeted approach was evaluated via MTT assay. The 10,000 cells/well of BT474 and MCF-7 cells were cultured in the 96-well plate for 24 h before treatment. The cells were then treated with the Mcl-1 nioplexes (containing Mcl-1 siRNA at 1.4 μg/mL) and TZ at a low concentration (0.1 μg/mL) or high concentration (10 μg/mL) and incubated under normal conditions for 72 h. The treated media were removed, and fresh media containing MTT was added. The plates were then incubated under normal conditions for 2 h. The MTT media were removed, and the formazan crystals were dissolved by DMSO. Absorbance was measured at 550 nm using a microplate reader. The percentage of cell-growth inhibition was calculated by Equation (2), and the siNT nioplex-treated group was used as a control.

### 2.8. Analysis of Dual Therapy by CompuSyn Software

The combination effect of Mcl-1 siRNA and TZ in non-constant ratio combinations was investigated in BT474 cells via MTT assay. The experiment included two groups: (1) a control group with untreated cells and individual drug treatments; and (2) a combination group with Mcl-1 siRNA and TZ at the concentration ratios of 0.1:1.4 and 10:1.4 (i.e., the concentration of Mcl-1 siRNA was fixed at 1.4 µg/mL). CompuSyn version 1.0 software (published and distributed by ComboSyn, Inc., Paramus, NJ, USA) was used to calculate the fractional effect (Fa) from the percentage growth inhibition at different drug concentrations. Fa = 0 represents no effect (100% cell viability or no inhibition); Fa = 1 indicates a complete effect (0% cell viability or total inhibition). The Chou–Talalay method was applied for combination analysis to determine the confidence interval (CI) value for each data point. The CI value quantifies the interaction between the drugs in the combination and indicates whether the effect is synergistic (CI < 1), additive (CI = 1), or antagonistic (CI > 1). Additionally, the dose reduction index (DRI) was calculated as the ratio of the dose of each drug used in combination with the dose of that drug when administered alone to achieve the same Fa level. A DRI value > 1 suggests that the dose of the drug can be reduced in combination therapy without compromising the desired therapeutic effect [50].

### 2.9. mRNA Expression Level Determination by RT-qPCR

The Mcl-1 gene silencing efficiency in BT474 was evaluated by reverse transcription polymerase chain reaction (RT-PCR) after single and dual treatment for 48 h. The mRNA was extracted and reverse transcripted to cDNA using the SuperPrep™ II Cell Lysis and RT Kit for qPCR (Toyobo, Osaka, Japan). Quantitative real-time PCR was quantified using the Thunderbird™ SYBR^®^ qPCR Mix (Toyobo, Osaka, Japan) on a LightCycler^®^ 480 Instrument II (Roche, Basel, Switzerland). The specific primers for glyceraldehyde 3-phosphate dehydrogenase (GAPDH) and Mcl-1 were designed from NM_001357943.2 and NM_021960.5, respectively. The GAPDH forward primer was TTTTGCGTCGCCAGCCG. The GAPDH reverse primer was CGCCCAATACGACCAAATCC. The Mcl-1 forward primer was GGA-GACCTTACGACGGGTT. The Mcl-1 reverse primer was AGTTTCCGAAGCATGCCTTG. The mRNA expression level was calculated by comparing the threshold cycle of Mcl-1 with GAPDH. The relative expression quantity was reported by comparing the targeted group with the untreated group.

### 2.10. Cell Death and Apoptosis Determination

The preliminary cell-death determination for the dual therapy was performed using a double-staining method and observed under a fluorescence microscope. BT474 cells were seeded at the cell amount of 10^4^ cells per well in a 96-well plate and cultured in a normal culture environment for 24 h prior to treatment. The cells were treated with a single or dual treatment for 24 h, and then the cells were stained with 10 µg/mL Hoechst 33342 and 0.5 µM SYTOX™ green nucleic acid stain for 30 min. The dead cells were inspected under an inverted fluorescence microscope using the untreated cells as a control.

Apoptosis was investigated using a flow cytometer. Briefly, the BT474 cells were cultured in 48-well plates at a density of 3 × 10^4^ cells per well and incubated overnight under normal conditions. The cells were then treated with 0.1 μg/mL TZ, 10 μg/mL TZ, siNT nioplexes, Mcl-1 nioplexes, or dual therapy. After 24 h of treatment, the cells were washed twice with DMEM without serum and trypsinized with 0.05% trypsin-EDTA. The cell suspension was then centrifuged at 1000 rpm for 5 min, and the cell pellets were rinsed with PBS. The cell pellets were then resuspended in 100 µL 1X Annexin V Binding Solution and stained with Annexin V, Alexa Fluor™ 647 conjugate, and 0.5 µM SYTOX™ green for 15 min under light protection conditions. The stained cells were diluted with a binding buffer before being analyzed with a flow cytometer.

### 2.11. Statistical Analysis

The experiments were performed in triplicate, and the data were presented as mean ± SD. Statistical analysis was performed using Microsoft^®^ Excel 2016 with 95% or 99% CI. Significant differences were declared at *p* < 0.05 or *p* < 0.01.

## 3. Results

### 3.1. Niosome Preparation and Characterization

#### 3.1.1. Niosome Characteristics

**Figure 2:**
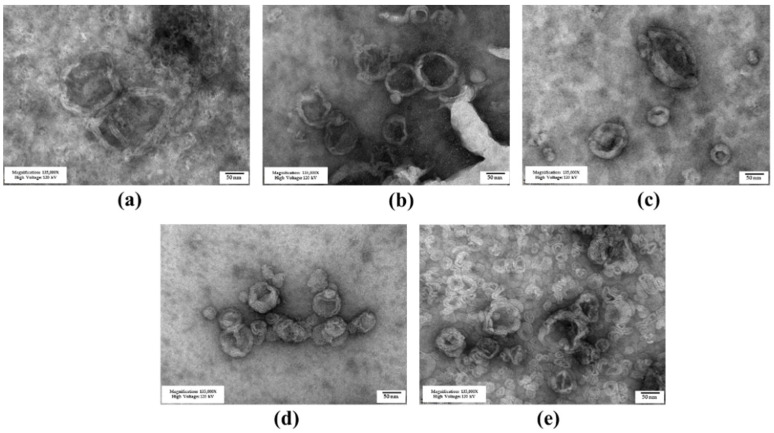
Transmission electron microscope images of cationic niosomes containing different cationic lipids; (**a**) 6as1 at a molar ratio of 1.5; (**b**) 6as1 at a molar ratio of 2.5; (**c**) 6as1 at a molar ratio of 3.5; (**d**) 4as1 at a molar ratio of 2.5; and (**e**) 8as1 at a molar ratio of 2.5. The scale bar represents 50 nm.

The niosomes containing different cholesterol-based cationic lipids were successfully prepared via the thin-film method with probe sonication. The molar ratio of the cholesterol-based cationic lipid to Span20 was first prepared at 2.5:2.5 according to a previous report, which showed good properties for nucleic acid delivery [51,52]. The size and surface-charge characteristics of the cationic niosomes are provided in Table 1. The hydrodynamic size of the niosomes prepared from 6as1 had the smallest size with a low polydispersity index (PDI) compared with other lipids at the same molar ratio. The zeta potential of all formulations revealed the positive charge on their surface, as expected for the successful self-assembly of the complexes with siRNA. The 4as1 niosome had the lowest charge, and the 6as1 niosome exhibited the highest positive charge. An increase in the cationic lipid amount from the 6as1:Span20 molar ratio of 1.5:2.5 to 3.5:2.5 resulted in a decreased zeta potential. The morphology examination of the prepared niosomes by TEM is provided in Figure 2. The images show that the prepared niosomes formed into nanosized multilamellar spherical vesicles.

#### 3.1.2. Niosome Stability 

**Figure 3:**
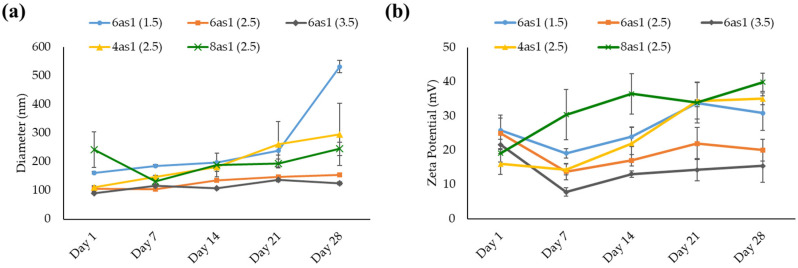
Hydrodynamic size (**a**) and zeta potential (**b**) for stability testing of niosomes at the designated time for 28 days.

The prepared niosomes were kept in a refrigerator (2–8°C), and the size and zeta potential were measured at the designated time for 28 days to evaluate their stability. The size of all niosome formulations generally increased with storage time (Figure 3a). The niosomes containing 6as1 at the lipid:Span20 molar ratio of 2.5:2.5 (6as1(2.5)) and 3.5:2.5 (6as1(3.5)) showed minimal change in particle size with low PDI compared to other formulations. The zeta potential of the niosomes was persistently positive during storage. The zeta potential of niosomes containing 4as1, 8as1, and 6as1 at the lipid:Span20 molar ratio of 1.5:2.5 (6as1(1.5)) tended to increase (Figure 3b). The increase in both the size and charge of the niosomes might be due to the aggregation or fusion of the vesicles [53].

### 3.2. Cellular Uptake in Breast Cancer Cells by Flow Cytometry and Fluorescence Microscope

**Figure 4:**
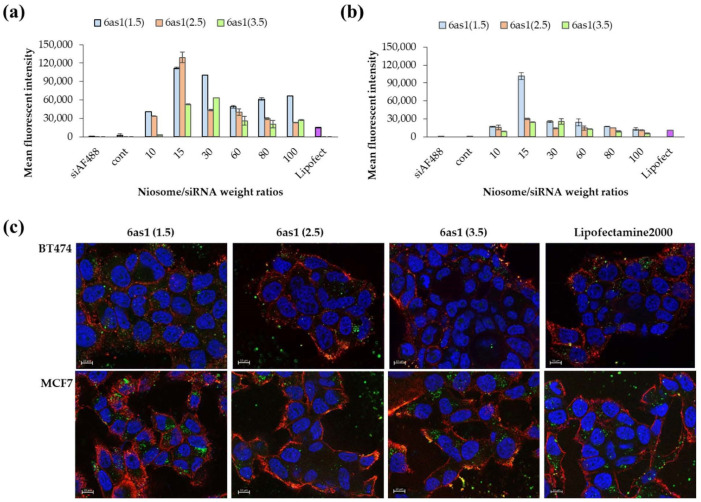
Cellular uptake abilities of 6as1 nioplexes at different niosome/siRNA weight ratios over 24 h as analyzed by flow cytometer in (**a**) BT474 cells and (**b**) MCF-7 cells. The data are presented as the mean fluorescent intensity ± SD from triplicates. (**c**) The fluorescence images of BT474 and MCF-7 cells transfected with 6as1/siAF488 nioplexes (green) for 24 h. Cells were stained with wheat germ agglutinin tetramethylrhodamine conjugate (red) and Hoechst 33342 (blue) before the images were taken. The scale bars are 10 μm.

The cellular uptake abilities of the nioplexes were examined in HER2-positive breast cancer cells (BT474) and HER2-negative breast cancer cells (MCF-7) via flow cytometry. From the preliminary uptake study of different niosome formulations, the niosomes prepared from 6as1 displayed the highest cellular uptake abilities and were comparable with Lipofectamine2000^®^ in both BT474 and MCF-7 cell lines (Figure S1). Therefore, the 6as1 niosome was chosen for further studies, and we observed the effect of the number of cationic lipids on cellular uptake abilities. In BT474 cells, the 6as1(1.5) and 6as1(2.5) nioplexes showed higher mean fluorescent intensity than the other nioplexes (Figure 4a). The cellular uptake ability depended on the niosome/siRNA weight ratios. The MFI was obtained at the weight ratio of 15. The cellular uptake abilities of the nioplexes in MCF-7 cells are shown in Figure 4b. The nioplexes of 6as1(1.5) had the highest MFI at the niosome/siRNA weight ratio of 15. In addition, the cells that were transfected with 6as1 nioplexes had an almost 100% positive fluorescent population (Figures S1 and S2). Therefore, the niosomes containing 6as1 could satisfactorily deliver siRNA into breast cancer cells.

The cellular uptake abilities of the 6as1 nioplexes were confirmed by fluorescence imaging (Figure 4c). The images indicate that the 6as1 nioplexes were taken up by both BT474 and MCF-7 cell lines, which is in accordance with our flow cytometry results. Most of the nioplexes were internalized and accumulated in the cytosol, where they were used as an active siRNA site to cleave the targeted mRNA.

### 3.3. Cell-Growth Inhibition of Mcl-1 siRNA Nioplexes and Mcl-1 siRNA Nioplex/TZ Dual-Targeted Therapy

#### 3.3.1. Cell-Growth Inhibition of Mcl-1 siRNA Nioplexes

**Figure 5:**
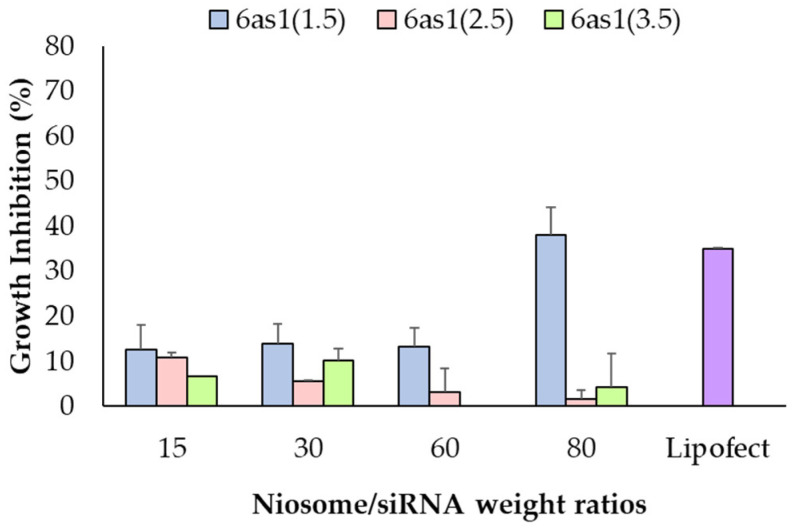
Cell-growth inhibition of Mcl-1 siRNA nioplexes prepared from different 6as1 niosomes at different niosome/siRNA weight ratios for 72 h in BT474 cells analyzed via MTT assay.

Optimum cell-growth inhibition in Mcl-1 siRNA single therapy was evaluated in BT474 cells via MTT assay. The 6as1 niosomes were selected to deliver the Mcl-1 siRNA into the cells because it displayed higher cellular uptake abilities compared to the other formulations. Maximum cell-growth inhibition was achieved at 37.95% ± 6.12% by 6as1(1.5) niosome at the niosome/siRNA weight ratio of 80, which was comparable with Lipofectamine2000^®^ (*p* > 0.05; Figure 5). Therefore, the niosomes containing 6as1 at the 6as1:Span20 molar ratio of 1.5:2.5 were selected for Mcl-1 siRNA delivery in further dual-targeted therapy.

#### 3.3.2. Cell-Growth Inhibition of Mcl-1 siRNA Nioplex/TZ Dual-Targeted Therapy and Synergistic Analysis

**Figure 6:**
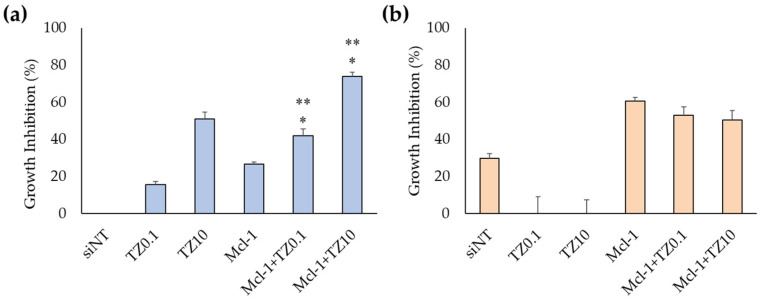
Cell-growth inhibition of (**a**) BT474 cells and (**b**) MCF-7 cells as determined by MTT assay after 72 h of treatment. The asterisk (*) represents significant differences between dual therapy and Mcl-1 nioplexes, and ** represents significant differences between dual therapy and TZ at the same concentrations (*p* < 0.05).

The suitable TZ concentrations were evaluated in BT474 cells for the dual-targeted therapy. The MTT results showed that the IC50 of TZ in BT474 cells was 0.69 μg/mL (Figure S3). Therefore, the TZ concentration for dual-targeted therapy was selected to be 0.1 μg/mL (TZ0.1) and 10 μg/mL (TZ10), which inhibited cell growth at 36.17% ± 8.36% and 54.45% ± 3.83%, respectively. Dual-targeted therapy was performed in both BT474 and MCF-7 cells (Figure 6a,b). The treatment of Mcl-1 nioplex/TZ dual therapy significantly increased cell-growth inhibition at 41.7% ± 3.6% in the Mcl-1 nioplex/TZ0.1 group and 73.8% ± 2.1% in the Mcl-1 nioplex/TZ10 group compared to the treatment of only Mcl-1 nioplexes or TZ. Cell-growth inhibition mainly resulted from the Mcl-1 nioplex treatment, which contrasted with the MCF-7 results. TZ did not affect cell growth, and dual therapy did not improve growth inhibition.

CompuSyn software was used to analyze the synergistic, additive, and antagonist effects of the dual-target therapy involving Mcl-1 siRNA and TZ in BT474 cells (Table 2). The combined effect of siNT and TZ (siNT/TZ) was also investigated as a negative control. The CI values of siNT/TZ0.1 and siNT/TZ10 were > 1, indicating that the combination was less effective than each drug administered independently and that they might interfere with each other’s actions. Mcl-1 siRNA combined with TZ at 0.1 μg/mL showed nearly additive effects (CI = 1.04), suggesting that the combined effect was equal to the effects of the individual drugs; however, the higher dose of TZ (10 µg/mL) in combination with Mcl-1 siRNA resulted in a CI of 0.57, indicating a synergistic effect. This implies that the dual treatment of Mcl-1 nioplexes and TZ enhanced the overall response and was more effective than a single treatment. The dual treatment of Mcl-1 nioplex/TZ10 allowed for significant reductions in the individual drug dosages by 28.35 (TZ) and 1.89 (Mcl-1 siRNA) times while still achieving Fa levels of 0.74. In contrast, higher dosages were required when each drug was used alone to achieve the same Fa level. This indicates that the combination therapy was more efficient, reduced drug dosage, and potentially minimized side effects.

### 3.4. mRNA Expression Level Determination by RT-qPCR

**Figure 7:**
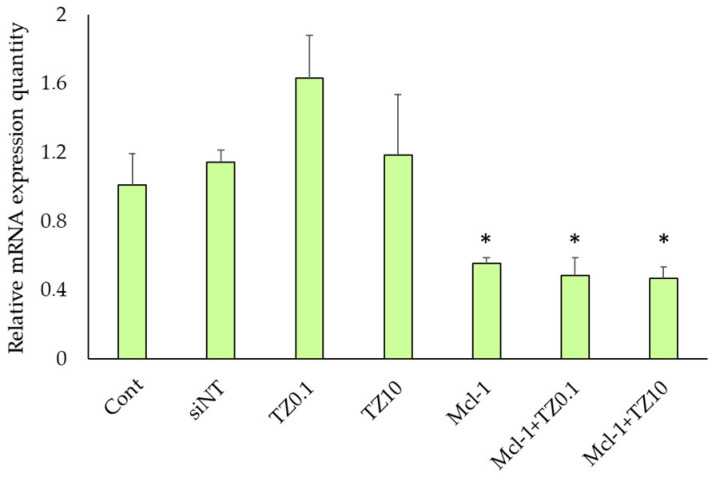
Relative Mcl-1 mRNA expression level in BT474 cells after 48 h of treatment with single and dual therapies as quantified by RT-qPCR. * represents significant differences from siNT nioplex transfection at *p* < 0.01.

The Mcl-1 mRNA expression levels of BT474 cells after treatment with siNT nioplexes, TZ at 0.1 μg/mL, TZ at 10 μg/mL, Mcl-1 nioplexes, and the dual therapy of Mcl-1 nioplex/TZ were determined by RT-qPCR. The mRNA expression levels were normalized with the untreated cells as a control group and calculated as a relative mRNA expression quantity (Figure 7). The significant depletion of Mcl-1 mRNA expression was observed at approximately 50% in the Mcl-1 nioplex transfected groups (*p* < 0.01), whereas TZ did not reduce mRNA levels. The dual therapy of Mcl-1 nioplex/TZ did not affect the mRNA level of Mcl-1 compared to the single Mcl-1 nioplex therapy.

### 3.5. Cell Death and Apoptosis Determination

**Figure 8:**
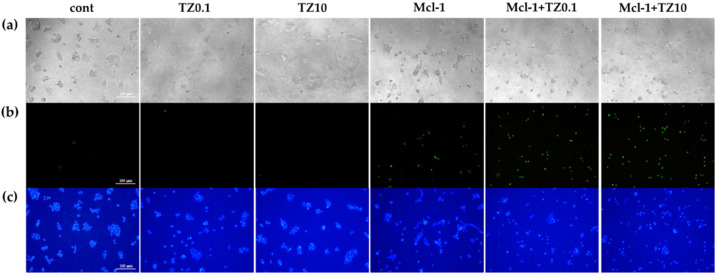
Cell death and apoptosis determination of BT474 cells stained with Hoechst 33342 and SYTOX^TM^ Green under a fluorescence microscope (100×) after 24 h of the single or dual treatments; (**a**) bright field; (**b**) green fluorescence; and (**c**) blue fluorescence.

The cell death and apoptosis of BT474 cells after treatment with the individual therapies or the dual therapy were preliminary examined via double staining with Hoechst 33342 and Sytox^TM^ Green and were investigated under an inverted fluorescence microscope (Figure 8). During apoptosis, chromatin condenses and cleaves into small fragments, but this does not occur in necrotic cells. Therefore, Hoechst 33342 can be permeabilized to stain DNA brighter in apoptotic cells than in normal cells [54]. Sytox^TM^ Green dye can stain the nuclei by penetrating the cell membrane of dead /necrotic cells but cannot cross the membrane of the live cells. The single TZ treatment at both concentrations resulted in apoptosis, as indicated by the brighter Hoechst 33342 DNA labeling compared to the untreated group. In contrast, the cells treated with Mcl-1 nioplexes were stained with both Hoechst 33342 and Sytox^TM^ Green, which indicates that the treated cells proceeded to apoptosis and cell death/necrosis after 24 h of transfection. The Mcl-1 nioplex/TZ dual therapy decreased cell viability compared to the single treatment. The bright field images revealed that the cells treated with TZ, Mcl-1 nioplexes, and the dual therapy had changed in cell morphology, and there was a decrease in cell density compared to untreated cells.

**Figure 9:**
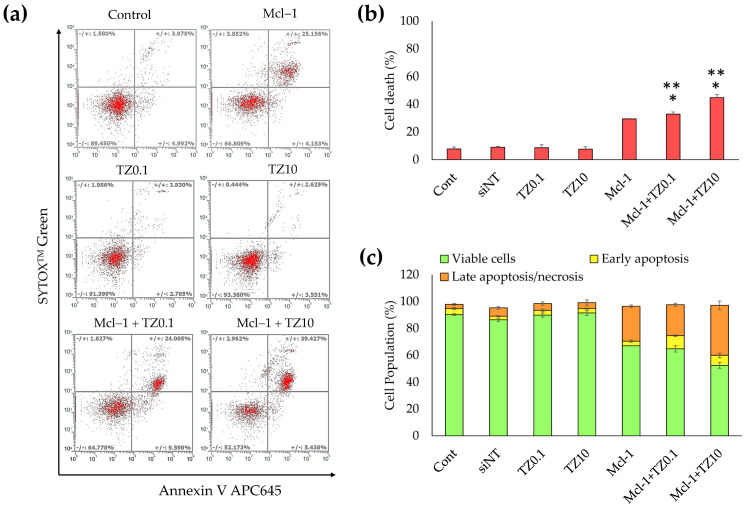
Determination of apoptosis in BT474 cells analyzed by flow cytometry. The BT474 cells were treated with the single treatments (TZ at 0.1 or 10 μg/mL, or Mcl-1 nioplexes) or the dual treatments (Mcl-1 nioplex/TZ0.1 or Mcl-1 nioplex/TZ10) for 24 h and stained with Annexin V-APC and SYTOX™ Green. (**a**) Scatter plots of each analysis. (**b**) The percentage of total cell death (the summation of apoptosis and necrosis). (**c**) The cell population percentages of the viable cells, early apoptosis cells, and late apoptosis/necrosis cells. The asterisk (*) indicates significant differences compared to the single treatment of TZ at the same concentrations and single Mcl-1 nioplexes (**) at *p*-value < 0.05.

Apoptosis and cell death were quantified by flow cytometry. BT474 cells were treated with a single treatment or dual treatment for 24 h, then analyzed by double staining with Annexin V-APC and SYTOX™ Green. The early apoptotic cells were stained with only Annexin V-APC, as shown in Q4 of Figure 9a, while the cells that progressed to late apoptosis or necrosis were stained with both Annexin V-APC and SYTOX™ Green (Q2 in Figure 9a). The total cell death in Figure 9b indicates that the single TZ treatment did not significantly increase apoptosis, whereas the single Mcl-1 nioplex treatment resulted in late apoptosis/necrosis at 29.47% ± 0.23%. The Mcl-1 nioplex/TZ dual treatment caused a remarkable increase in cell death compared to the single treatments (*p* < 0.05). The flow cytometry results showed that the cells treated with Mcl-1 nioplexes were predominantly in late apoptosis or necrosis (26.03% ± 1.01%). A similar cell-death mechanism was found in the dual treatment with the percentage of late apoptosis/necrosis at 23.07% ± 1.17% in the Mcl-1 nioplex/TZ0.1 treated group and at 37.38% ± 3.23% in the Mcl-1 nioplex-treated group (Figure 9c). These results concurred with our cell-growth inhibition result, which showed that the dual therapy of a high-concentration TZ and Mcl-1 nioplexes caused the lowest BT474 cell growth.

## 4. Discussion

Cationic niosomes were successfully formulated from Span20 and the different cholesterol-based cationic lipids (4as1, 6as1, and 8as1). The niosomes each exhibited a positive charge, which was important for binding with the negative charge of siRNA, and were nanosized, which was necessary for siRNA delivery. The multilamellar vesicles were observed in the TEM images, which agreed with the particle size of 100–1000 nm [55]. In the case of the 6as1 niosomes, the niosomes with the lower amount of cationic lipids showed higher zeta potential with higher SD compared to the niosomes containing higher amounts of cationic lipids; however, significant differences between the groups were not found (*p* > 0.05). This result might be due to the non-uniformity of cationic lipid incorporation in the discrete particles of the niosomes prepared from the lower lipid contents. In the 6as1 niosomes, the increase in cationic lipid amount led to a decrease in niosome size. Cholesterol has been typically used as the helper lipid in niosome formulations because it can increase the rigidity and mechanical strength of the bilayer, which leads to improved stability of the lipid bilayer [56,57] and a reduction in the aggregation of particles in the presence of serum [58]. In contrast with other conical cationic lipids, such as DOTMA or DOPE, cholesterol-based cationic lipids have a cylindrical molecular shape with a decreased membrane fusion ability compared to other cationic lipids [59]. This property may also enhance the size and charge stability of niosomes. In addition, the incorporation of the cholesterol structure in niosomes containing a hydrophilic nonionic surfactant (e.g., polysorbate) causes a decrease in surface energy and particle size due to an improvement in bilayer hydrophobicity [35]. Therefore, the cholesterol-based cationic lipids provided a positive charge on the niosome surface and enhanced the stability of the cationic niosomes.

The prepared niosomes were kept in a refrigerator at 4–8 °C throughout the experiments since the storage temperature affected their physicochemical properties and decreased transfection efficiency. High temperatures cause niosome aggregation due to the reduction of the positive surface charge and electrostatic repulsion [46,60]. The stability study indicated that all cationic niosomes were stable and able to be kept for at least 28 days. The niosomes containing 6as1 showed the lowest change in size and charge among all studied niosomes (Figure 3); however, the niosome size gradually increased with time.

In a previous study, lipid transfer correlated with liposome size, size distribution, and liposome composition. In the system with only one lipid, the average size change was time-independent, and the size distribution depended on the diffusion [61]. However, the cationic niosomes in this study were not composed of only cationic lipids; therefore, the amount of lipid in each particle may not have been precisely equal. Consequently, the size of the niosomes may have gradually increased due to the reduction in electrostatic repulsion and loss of structural rigidity during the lipid transfer process.

The 6as1 niosomes also showed the highest siRNA delivery levels into the breast cancer cell lines compared to the other niosomes (Figure S1). It has been reported that the different cationic ionic headgroups, linkers, and hydrophobic domains of cationic lipids affect different niosome properties, transfection efficiencies, and toxicity [46,62,63,64,65]. In this study, each cationic lipid had the same cholesterol hydrophobic domain and carbamate linker. The 6as1 niosomes contained the trimethylammonium (quaternary ammonium) headgroup cationic lipids and had an increased cellular uptake and size stability compared to the cationic lipids containing the tetramethylguanidinium headgroup (4as1 and 8as1). The benefit of the quaternary ammonium headgroup cationic lipids (e.g., DOTMA, DOTAB, CTAB, and DORIE) in nucleic acid delivery has been shown to be its high transfection efficiency in various cell types [63,66,67]. The guanidinium headgroup cationic lipids have also been proposed for nucleic acid delivery and incorporation in liposome or niosome formulations as they are efficient in DNA binding and have transfection abilities [68,69,70]; however, the direct comparison of the cationic niosomes prepared from the quaternary ammonium and guanidinium headgroups in siRNA delivery has not yet been clearly reported. Our results indicate that the tetramethylguanidinium headgroup in the 4as1 and 8as1 niosomes may have exhibited higher steric hindrance effects or electronic repulsion, which may be interfered with by the interaction of phosphate groups in siRNA compared to the trimethylammonium headgroup in the 6as1 niosomes [71,72]. In addition, the 6as1 niosomes displayed lower cytotoxicity (IC50 value > 500 μg/mL for 6as1(1.5) niosomes and 6as1(2.5) niosomes, and >300 μg/mL for 6as1(3.5) niosomes) compared to other niosomes (Figure S4).

By varying the cationic contents in the 6as1 niosomes, the 6as1(1.5) and 6as1(2.5) niosomes showed the highest siRNA delivery rates to breast cancer cells at the niosome/siRNA weight ratio of 15; however, the 6as1(1.5) niosome showed the wider niosome/siRNA weight ratio range for delivering siRNA to the cells (Figure 4a). Additional investigations into the size and zeta potential of the 6as1 nioplexes is provided in Figure S5. The zeta potential of the 6as1 nioplexes depended on the niosome/siRNA weight ratio, which was a negative charge at the niosome/siRNA weight ratios of less than 15. At the higher niosome/siRNA weight ratios, the siRNA was entirely complexed with the 6as1 niosomes, and the nioplex surface charge became positive at the niosome/siRNA weight ratio of 15. Interestingly, the cellular uptake study demonstrated that the 6as1(1.5) niosomes provided the best siRNA delivery in both the BT474 and MCF-7 cells, which indicates that this cationic niosome may be used for wider cell types. Therefore, the 6as1 niosomes were chosen for Mcl-1 siRNA delivery for the next experiments. Although the highest cellular uptake was achieved at the niosome/siRNA weight ratio of 15, the highest growth inhibition in BT474 cells was obtained by transfecting the 6as1(1.5) nioplexes at the niosome/siRNA weight ratio of 80. Since the nioplexes at the niosome/siRNA weight ratio of 15 had negative or neutral surface charges (Figure S5), it is possible that the siRNA dominance attached to the nioplex surfaces and did not incorporate into the lipid vesicles, which leads to siRNA degradation [73]. In addition, the large size of the 6as1(1.5) nioplexes at the niosome/siRNA weight ratio of 80 inhibits the effect of serum on transfection efficiency, facilitates cell membrane contact, and internalizes large intracellular vesicles, which were easily disrupted and released the siRNA from the nioplexes [74,75,76].

Mcl-1 overexpression is found in many cancers, including breast cancer. It acts as an anti-apoptotic protein that blocks apoptosis and enables cancer cells to evade cell-death signals [77]. It has been reported that there is a positive correlation between a high level of Mcl-1 and HER2 expression [78]. The suppression of Mcl-1 increases the sensitivity of HER2 inhibitors, such as Lapatinib, in HER2 overexpression breast cancer cells [79,80]. TZ specifically targets the overexpressed HER2 receptor in HER2-positive breast cancer to hinder its signaling pathways and disrupt cancer cell proliferation [81]. The combination of TZ and Mcl-1 inhibitors results in a synergistic effect and a more comprehensive and potent approach to cancer treatment [82]. Therefore, this study approached breast cancer treatment with a dual-targeted therapy targeting HER2-mediated signaling (TZ treatment) and Mcl-1 mRNA (Mcl-1 nioplex treatment). The cell-growth inhibition of TZ single treatment in BT474 cells displayed a dose-dependent response, and the IC50 value (0.69 μg/mL) was approximately equal to that of previous reports [83,84,85]. The Mcl-1 nioplex/TZ dual therapy significantly increased cell death, with an increased cell-growth inhibition achieved using TZ at 10 μg/mL (Figure 6). The combination analysis by the CompuSyn software revealed a CI value of 0.53 at Fa 0.74, which was a significant synergistic effect between the Mcl-1 nioplexes and TZ [86,87]. The dual therapy exhibited dose reduction compared to the administration of individual treatment to achieve the same level of efficacy. The prospect of lower doses might minimize possible adverse effects that enhance patient tolerance and be advantageous in treating drug-resistant HER2-positive breast cancer. Our results are in accordance with a previous study that indicated the synergistic effects of the TZ and Mcl-1 siRNA combination [88]. In addition, the RT-qPCR results revealed that the depletion of Mcl-1 mRNA expression was observed in both the Mcl-1 nioplex single treatment and the dual treatment, which indicates successful cell-growth inhibition via the knockdown of Mcl-1 mRNA (Figure 7). Conversely, TZ was not sensitive to MCF-7 cells, and the Mcl-1 nioplex/TZ dual therapy did not promote cell-growth inhibition since MCF-7 cells have very limited HER2 expression [89]. The apoptosis assay also confirmed the increased cancer cell death via the Mcl-1 nioplex/TZ dual treatment, which proceeded to late apoptosis/necrosis after 24 h of treatment. The single TZ treatment did not show any significant apoptosis effects in this study. This may be due to the low TZ concentration used and the duration of the treatment, which were not enough to exhibit the apoptosis response [90]. Our findings suggest that dual-targeted therapy by Mcl-1 nioplexes and TZ is a potential strategy for the future progress of breast cancer treatments in HER2 overexpression patients.

## 5. Conclusions

The preparation of novel lipid-based nanoparticles (cationic niosomes) from cholesterol-based cationic lipids and Span20 were successful. The different hydrophilic headgroups of cationic lipids affected niosome properties and transfection efficiencies in breast cancer cells. The siRNA nioplexes targeted in the Mcl-1 mRNA exhibited cell-growth inhibition in both HER2-positive and HER2-negative breast cancer cells. The dual treatment of Mcl-1 nioplexes and TZ significantly decreased cell survival and promoted apoptosis compared to their single treatment in HER2-overexpression breast cancer cells. The synergistic interaction between Mcl-1 nioplexes and TZ was confirmed by CompuSyn software. Consequently, the dual therapy targeting Mcl-1 and HER2 may be a potential treatment regimen for HER2-overexpression breast cancer.

## Figures and Tables

**Table 1:** Hydrodynamic size and zeta potential of the niosomes.

Formulations (Molar Ratios)	Hydrodynamic Size		Zeta Potential (mV) ± SD
	Diameter (nm) ± SD	PDI ± SD	
6as1 (1.5:2.5)	161.6 ± 4.7	0.3 ± 0.02	25.8 ± 4.4
6as1 (2.5:2.5)	105.8 ± 9.6	0.3 ± 0.06	24.9 ± 4.5
6as1 (3.5:2.5)	90.8 ± 3.3	0.4 ± 0.03	21.6 ± 1.5
4as1 (2.5:2.5)	110.9 ± 2.8	0.5 ± 0.01	16.0 ± 3.1
8as1 (2.5:2.5)	242.3 ± 62.0	0.6 ± 0.13	19.1 ± 2.6

**Table 2:** The combination analysis data for dual therapy by CompuSyn software.

Drug Combo (μg/mL)				Dose Reduction Index (DRI)		Doses of Each Drug Required to Achieve Specific Fa Level	
TZ	Mcl-1 siRNA	Fa	CI	TZ	Mcl-1 siRNA	TZ	Mcl-1 siRNA
0.1	1.4	0.42	1.04	13.92	1.04	1.39	1.45
10	1.4	0.74	0.57	28.35	1.89	283.48	2.64

## Data Availability

The data presented in this study are available in this article.

## Supplementary Materials

The following supporting information can be downloaded at: https://www.mdpi.com/article/10.3390/pharmaceutics15102424/s1, Figure S1: Cellular uptake of 4as1, 6as1, and 8as1 nioplexes at different niosome/siRNA weight ratios for 24 h analyzed by flow cytometer in (a) mean fluorescent intensity in BT474 cells, (b) mean fluorescent intensity in MCF-7 cells, (c) fluorescent positive population in BT474 cells and (d) fluorescent positive population in MCF-7 cells; Figure S2: Fluorescent positive population after transfecting cells with 6as1 nioplexes at different niosome/siRNA weight ratios analyzed by flow cytometer (a) BT474 cells and (b) MCF-7 cells; Figure S3: Dose response curve of trastuzumab in BT474 cells; Figure S4: Cytotoxicity and IC50 values of cationic niosomes with different cationic lipids in BT474 cells after 72 h of the treatment, analyzed by MTT assay; Figure S5: Hydrodynamic size and zeta potential of siRNA nioplexes prepared from 6as1(1.5), 6as1(2.5) and 6as1(3.5) niosomes analyzed by Zetasizer Nano ZX.
