# Supplementary material for: Dual-Targeted Therapy in HER2-Overexpressing Breast Cancer with Trastuzumab and Novel Cholesterol-Based Nioplexes Silencing Mcl-1
Source: Pharmaceutics. 2023 Oct 4;15(10):2424. doi: 10.3390/pharmaceutics15102424 (PMC10610066; doi:10.3390/pharmaceutics15102424)
Supplement: Supplementary file 1 [file pharmaceutics-15-02424-s001.zip › pharmaceutics-2611440-supplementary.pdf]

Supplementary Materials

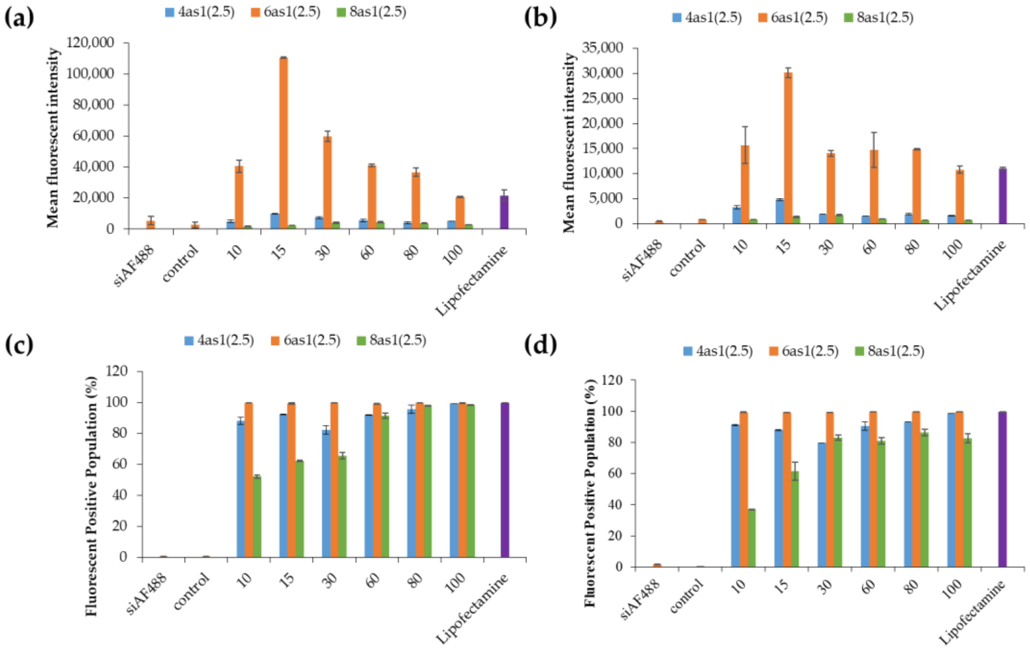

Figure S1: Cellular uptake of 4as1, 6as1, and 8as1 nioplexes at different niosome/siRNA weight ratios for 24 hr analyzed by flow cytometer in (a) mean fluorescent intensity in BT474 cells, (b) mean fluorescent intensity in MCF-7 cells, (c) fluorescent positive population in BT474 cells and (d) fluorescent positive population in MCF-7 cells.

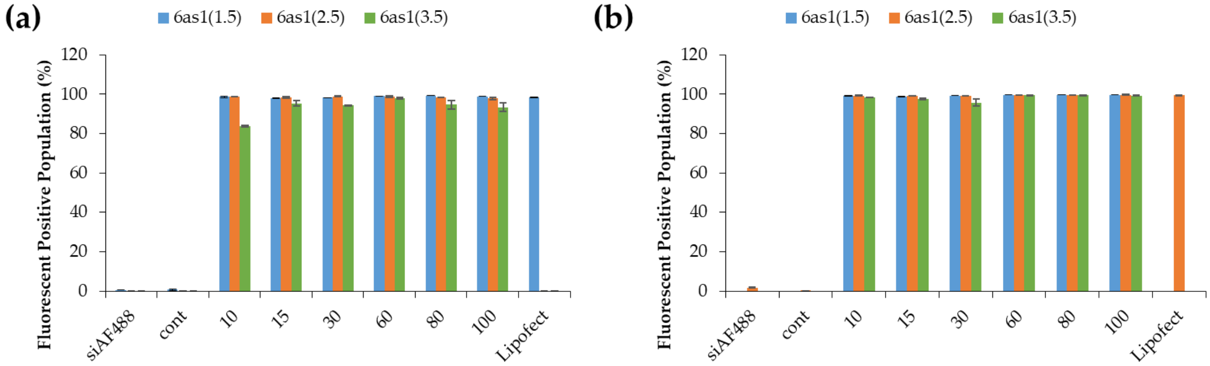

Figure S2: Fluorescent positive population after transfecting cells with 6as1 nioplexes at different niosome/siRNA weight ratios analyzed by flow cytometer (a) BT474 cells and (b) MCF-7 cells.

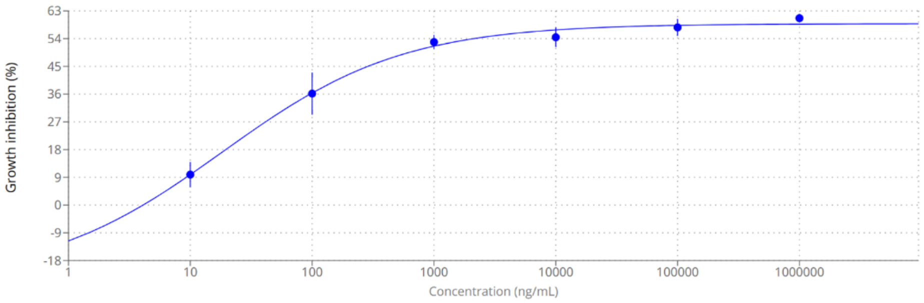

Figure S3: Dose response curve of trastuzumab in BT474 cells.

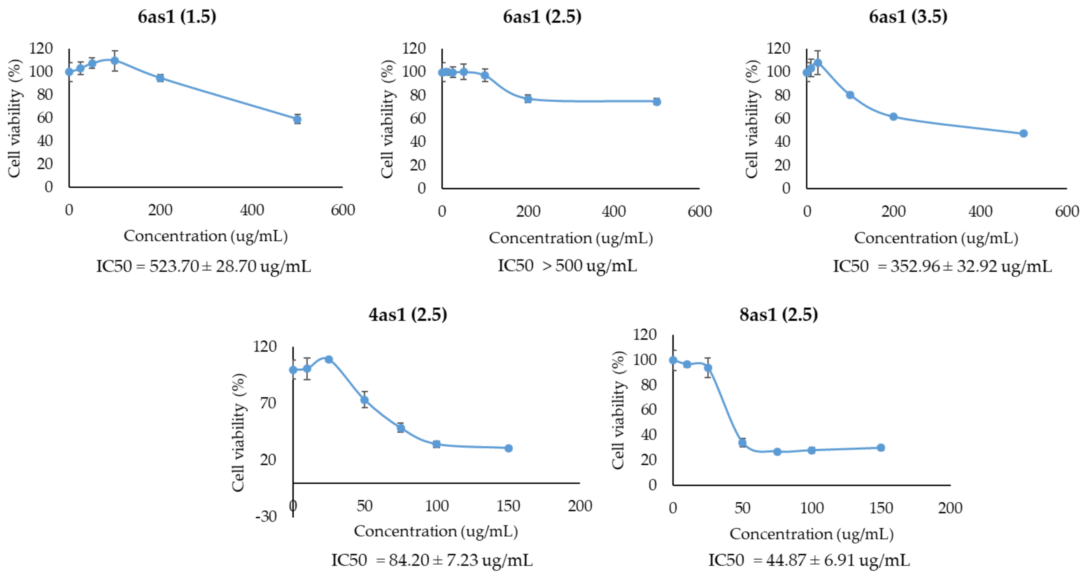

Figure S4: Cytotoxicity and IC50 values of cationic niosomes with different cationic lipids in BT474 cells after 72 hr of the treatment, analyzed by MTT assay.

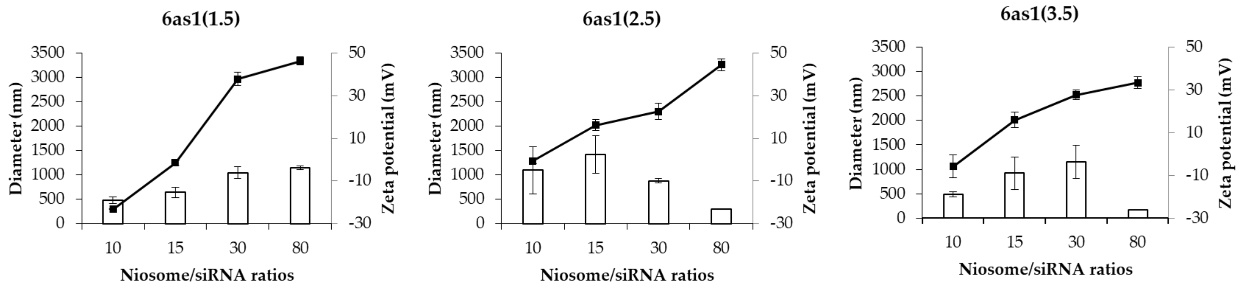

Figure S5: Hydrodynamic size and zeta potential of siRNA nioplexes prepared from 6as1(1.5), 6as1(2.5) and 6as1(3.5) niosomes analyzed by Zetasizer Nano ZX.
